# Spatial pattern of herbaceous seed dispersal by ungulates in grasslands of Doñana, SW Spain

**DOI:** 10.1371/journal.pone.0327616

**Published:** 2026-03-09

**Authors:** María José Leiva, Jose María Fedriani

**Affiliations:** 1 Departamento de Biología Vegetal y Ecología, Universidad de Sevilla, Sevilla, Spain; 2 Centro de Investigaciones sobre Desertificación CIDE, CSIC-UVEG-GV, Carretera de Moncada a Náquera, Moncada (Valencia), Spain; 3 Estación Biológica de Doñana (EBD - CSIC), Seville, Spain; University of Minnesota, UNITED STATES OF AMERICA

## Abstract

Most research on endozoochorous seed dispersal by ungulates emphasizes long-distance dispersal and its dependence on ungulate species and habitat heterogeneity. In contrast, the role of ungulate community composition in shaping local (around one hundred hectares or less) spatial patterns in the distribution of plant seeds remains largely unexplored. To address such key question, we quantified ungulate seed dispersal at two grasslands in the Doñana National Park (southwestern Spain). One grassland (Martinazo) was used by a mixed community of four ungulate species (deer, wild boar, cattle, and horses), while the other one (Matasgordas) was used almost exclusively by deer. At each site, a plot was established, and ungulate fecal samples were systematically collected and georeferenced from early spring to mid-summer. The seeds contained in the samples were extracted and identified in the laboratory. Our results revealed clear differences between sites in the total number of dispersed herbaceous seeds (1,302 and 606 seeds in Martinazo and Matasgordas respectively) and the most frequently dispersed plant families. Within the four-species community, cattle and deer differed most in the taxonomic composition of the seeds they dispersed, suggesting that herbivore-specific seed selection and dispersal act as key drivers of grassland structure at fine spatial scales, mirroring dynamics typically observed at broader scales. Moreover, only in the four-ungulate community did we detect significant spatial patterns in seed dispersal. These included positive effects of short-distance feces aggregation, spatial covariance in seed content among nearby feces, and a strong correlation between seed content and local feces density. Seed families predominantly dispersed by cattle also exhibited significant spatial structuring. These findings have important implications for biodiversity management in semi-natural and protected ecosystems, as they demonstrate that the management of ungulate species (both wild and domestic) also influences plant communities. This highlights the need to consider the functional differences among herbivores in ecosystem conservation and restoration strategies.

## 1. Introduction

Large herbivorous ungulates are important seed dispersers through both endozoochory and epizoochory. Thus, these dispersers contribute to the formation of plant seed banks, particularly for herbaceous species [1,2], playing thus a key role in shaping herbaceous plant communities [3–6]. The guild of ungulate species coexisting in an area has been classically regarded as exhibiting high functional overlap in terms of seed dispersal [7], because most large ungulates consume a wide variety of herbaceous plants [3,8,9]. However, more recently, complementarity in seed dispersal has also been observed among sympatric ungulates [10]. For instance, Picard et al. [11] found that in agro-forested landscapes, the feces of red deer (*Cervus elaphus*) contained greater seed numbers and species richness than those of sympatric wild boar (*Sus scrofa*) and roe deer (*Capreolus capreolus*). Polak et al. [12] also identified complementarity in seed dispersal by two reintroduced wild ungulates (the Arabian oryx, *Oryx leucoryx*, and the Asiatic wild ass, *Equus hemionus*) in the Negev Desert, where another ungulate, the dorcas gazelle (*Gazella dorcas*), has historically been present. In this case, the three species of ungulates dispersed different assemblages of plant species. Specific traits that have been discussed as the cause of differential endozoochorous seed dispersal among ungulates are the gut passage time which varies between co-ocurring ungulates [13], the differential animal selectivity on sward plants for instance between sheep and cattle [14] linked to body physical characteristic (i.e., smaller mouth and more mobile lips in sheep) and, at a larger scale, the habitat selectivity by the ungulate disperser [11].

Ungulates are key long-distance seed dispersers [2,5,15]. Thus, their role in maintaining habitat connectivity through seed dispersal in fragmented landscapes [2,6,16,17] and the differences in habitat selection and plant dispersal among ungulate species have frequently been documented, particularly in large areas composed of a mosaic of land uses and vegetation types [11,15]. Studies of seed dispersal by ungulates at medium and small spatial scales have also been conducted in recent decades, focusing mainly on the degree of convergence between local flora and dispersed seeds [6], temporal changes in seed dispersal within local herbaceous communities [18], or differences among local ungulates in the pool of dispersed seeds [19], among others. However, studies on the local spatial patterns of seed dispersal mediated by ungulate herbivores in grassland communities remain much scarcer (but see [20]). Nonetheless, they are crucial, because fine-scale recruitment influences local population and community dynamics [21] and short-distance seed dispersal plays a key role in shaping the potential area of plant recruitment and subsequent ecological processes such as competition, mating, and predation [5].

In the last decades, wild ungulate populations have experienced significant density increases in many areas of Europe due to the reduction of natural predators and changes in management practices (e.g., supplementary feeding) [22]. Additionally, wild ungulates often coexist with domestic ungulates in many human-managed grazing areas [18,23–25], including protected areas where domestic species may still persist as remnants of past land use [26,27]. However, the effects of ungulate community composition on seed dispersal and spatial pattern generation at the local grassland scale remain largely understudied.

In this study, we assess the dispersal of herbaceous seeds by two ungulate communities within protected Mediterranean grasslands in Doñana National Park (southwestern Spain). This area is recognized as a World Heritage Site and is one of Europe’s most important strongholds for biodiversity conservation [28] because of its wide range of habitats; a diverse combination of European and African flora and fauna, including many endemic species and several migratory waterbird populations which are globally threatened [29]. The area has been the focus of extensive research on seed dispersal mediated by birds and frugivorous mammals [30–34], frequently at the scale of single plant populations [35]. However, very few studies have addressed seed dispersal by ungulate herbivores in the park’s grassland ecosystems, where numerous herbaceous species may be dispersed endozoochorously under the so-called “foliage is the fruit” hypothesis [3]. The objectives of this study are *i*) to assess potential differences in the quantity and taxonomic composition of herbaceous seeds endozoochorously dispersed by ungulates in two grassland sites that differ in ungulate species richness and composition, as well as in the domestic or wild status of these ungulate communities, and *ii*) to investigate potential spatial patterns of herbaceous seed dispersal generated by these two communities, both in terms of total seed number and the most frequently dispersed seeds taxa. Based on findings from other European habitats [36], we hypothesize that the two ungulate communities will differ in both the amount and the taxonomic composition of dispersed seeds. Furthermore, we expect differences in the spatial pattern of seed deposition, reflecting variation in ungulate size and behavior. Species such as cows, which tend to produce aggregated feces, would also be more likely to generate aggregation of dispersed seeds [20,21].

## 2. Materials and methods

### 2.1. Study area and sites description

The study was conducted in Doñana National Park, located in southern Spain (510 km²; 37º 10′ N; 6º 26′ W) in a flat area ranging from 0 to 80 m above sea level [37]. The climate is Mediterranean, with wet, mild winters and long, dry summers. The mean annual temperature is 17.7ºC, while annual rainfall averages 465.6 ± 34.5 mm, although it varies considerably between years. Rainfall predominantly occurs from September to May and is scarce from June to August (data from [38]). The Doñana landscape is humanized and fragmented, with patches of woody vegetation frequently isolated by croplands, grasslands, marshes, dunes, and urban areas.

In the contact zone between scrubland and marshland lies a transitional area predominantly composed of grassland, where this study was conducted. This ecotone, locally called “la vera”, varies considerably in width (from a few meters to several hundred) and is intensively grazed by wild and domestic ungulates [39–41]. The ecotone is associated with a gradient of environmental conditions (such as water-table depth and vulnerability to temporal flooding [42] that drives variation in the herbaceous community. Two main grassland types can be distinguished: dry grassland and wet grassland. The former occurs on sandy soils and is typically free from winter flooding. Representative species include the annuals *Anthoxanthum ovatum* and *Vulpia membranacea*, the perennials *Cynodon dactylon* and *Panicum repens*, and isolated individuals of *Asphodelus ramosus* and *Armeria gaditana*. Wet grassland develops on clayey-sandy soils, barely raised above the marsh level, and is prone to episodic winter flooding. The perennial *C. dactylon* is also abundant here and frequently occurs alongside dense stands of the reed *Juncus maritimus*. This ecotone is functionally connected to adjacent habitats. For example, ungulates use the grasslands, the herbaceous gaps within scrubland, and the elevated areas of the marsh at different times of the year [42].

Ungulates commonly found in the grassland include red and fallow deer (*Cervus elaphus* and *Dama dama*), wild boar (*Sus scrofa*), domestic cow (*Bos taurus*) and horse (*Equus ferus caballus*). Their feeding habits vary seasonally [43]. Generally, ungulates predominantly consume herbaceous plants from late winter to early summer but shift their diet toward woody species from mid-summer to early winter, when grassland biomass is scarce ([44] and references herein).

**Fig 1:**
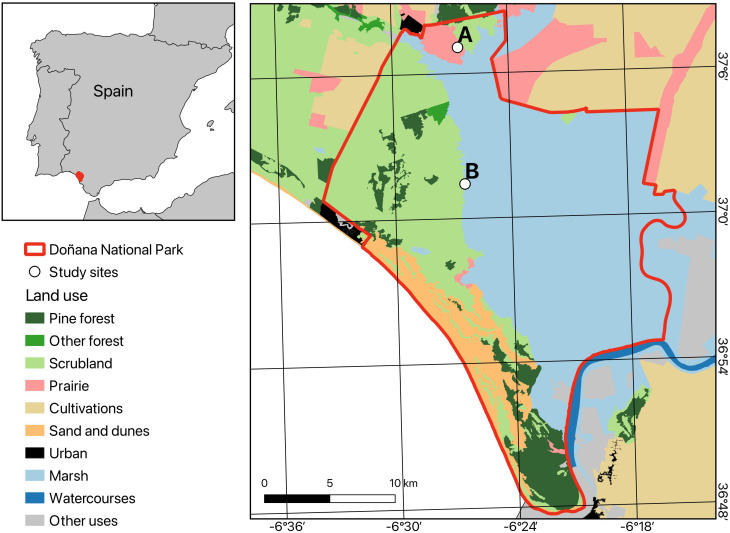
Doñana National Park, southwestern Spain, bordering the Atlantic coast. Red line = Park limit. White dots (A, B) indicate the location of the study plots at Matasgordas and Martinazo grasslands, respectively.

To conduct this study, two grassland sites within “la vera” were selected, called Martinazo (37° 1’ 24.46” N, 6° 26’ 24.89” W) and Matasgordas (37° 7’ 4.38” N, 6° 26’ 33.71” W), respectively (Fig 1). A permit was obtained from the park management office (ref: 2019107300002261/IRM/MDCG/mes). The sites are located approximately 10 km apart and each comprises both dry and humid grassland areas. Native vegetation at both sites has been historically altered [44]. In Matasgordas, a large pastureland with patches of Mediterranean scrub and scattered trees, was created in 1970 while in 1996, this area was classified as a strict nature reserve, and livestock were largely removed from the site. In Martinazo extensive livestock farming (cattle, horses, and pigs), have been practiced there since at least the 14th century [45]. The area was designated a Biological Reserve in 1964, which resulted in a reduction of livestock density. Today, Martinazo consists of a moderately extensive pastureland with a few sparse trees and scattered scrub patches. Livestock ranching (cattle and horses) still occurs there, although its intensity has diminished in recent decades [26]. Average deer density in Doñana National Park is 10.1 individuals per km^2^ being, at least, 2.2 times higher at Matasgordas than at Martinazo [44,46]. The two sites also differ in their physical characteristics which to some extent may affect the composition and abundance of grassland species. The topsoil at Martinazo contains approximately twice the amount of organic matter, phosphorus, and nitrogen compared to Matasgordas. Additionally, the groundwater table is shallower at Martinazo (2.39 m) than at Matasgordas (4.56 m) [26]. This condition makes the former area more vulnerable to episodic flooding, which in turn may affect the utilization of the pasture by ungulates.

At each site, a tetragonal plot was established for the collection of ungulate feces. The plot sizes were set to prevent overlap with adjacent scrubland and marshland. Thus, at Martinazo, where the grassland ecotone band is narrower, the plot measured 10 ha while at Matasgordas the plot measured 90 ha.

### 2.2. Feces sampling

Herbivore feces were sampled from early spring to mid-summer of 2020, corresponding to the fruiting season of most herbaceous species in the area [48]. Sampling followed the methodology described by Fedriani et al. [32]. A series of starting points were regularly distributed along one edge of each plot. From each starting point, a non-systematic zigzag transect was conducted toward a non-fixed point on the opposite edge of the plot, and the return path followed a different trajectory. This zigzag sampling procedure allows bypassing episodic flooding and facilitates the continuous sampling of grassland within each complete sector of the plot. During each survey (i.e., a complete transect across the plot and back), all intercepted ungulate fecal units (defined as any spatial aggregation of fecal material) were recorded (deposition points hereafter). The spatial coordinates of each point were logged using a Global Position System-reading. Feces were identified to the species level based on morphological characteristics. Deer feces (including both red and fallow deer) are 1–2.5 cm long and 0.8–1.4 cm wide, brownish-black, and of firm consistency; due to considerable overlap in size and shape, feces from both species were not reliably distinguishable and are hereafter referred to collectively as “deer.” This grouping may have influenced the results in some extent, given that the two species differ in certain habitat preferences [43]. Nevertheless, red deer predominate in both areas, accounting in 2014 for approximately 91% and 66% of the deer density in Matasgordas and Martinazo respectively [46]. Further, the abundance of fallow deer during the course of this study (spring and summer of 2020) was much lower (Authors *personal observations*). Wild boar feces consist of compact units approximately 5 cm in diameter, often forming larger masses, dark brown to black in color, and with heterogeneous content. Horse feces are oval, at least 4 cm thick, smaller than a tennis ball, greenish-brown, and oblong with rounded ends, occurring singly or in amorphous groupings. Cow feces are larger, of pasty consistency, and typically deposited in flat, circular piles [49,50].

In addition to feces recording, the presence or absence of woody vegetation associated to each deposition point was assessed to characterize the vegetation physiognomy of seed arrival microsites (e.g., [51]). A circular area with a 1-meter radius centered on each fecal unit was established, and the relative shrub cover was visually estimated using a subjective scale ranging (from 0% to 100%).

Samples were collected from each fecal unit (50 cm³ per sample, when possible) and stored in labeled paper envelopes indicating the herbivore species, site, transect code, and date. Samples were kept at room temperature in the laboratory of the University of Seville until seed extraction. A total of 231 fecal samples were collected—113 from Martinazo and 118 from Matasgordas—during 145 surveys (65 in Martinazo and 75 in Matasgordas) conducted over 39 field visits. One sample (designated S72) contained 2,614 seeds, representing 37% of the total recorded seeds. These seeds were almost exclusively from the genus *Juncus* (see S1 Table), which produces a large number of very small seeds. Due to its disproportionate contribution, this sample was excluded from all analyses to avoid skewing the results. This decision was based on Grubb’s test confirming that S72 was an outlier (G = 10.73; critical G at α 0.05 = 3.44; p < 0.001).

### 2.3. Seed identification and quantification

Approximately 2.5 g of dry weight (DW) per sample were used for seed extraction, except in cases with insufficient material (16% of samples, *n* = 37), where the entire sample was processed. The dry weight of each individual sample was recorded prior to processing. Samples were rehydrated and carefully washed using a series of sieves with mesh sizes of 0.5 mm, 0.1 mm, and 0.01 mm under running water. The retained material was then visually inspected under a stereo microscope (8 × –35 × , Leica EZ4) to detect the presence of seeds. Only unbroken, non-empty, and apparently intact seeds were selected and stored in Petri dishes for subsequent identification. The dry weight of each sample was then used to standardize seed content, expressed as the number of seeds per gram DW.

Seeds were identified to the highest possible taxonomic level using *Flora Ibérica* [47] and *Flora de Andalucía Occidental* [48]. Additionally, a reference seed collection, compiled during the study year from plant species occurring at the study sites, was consulted. Expert taxonomists also collaborated in identifying specific seed taxa, including Dr. Benito Valdés Castrillón and Dr. Ádám Lovas-Kiss.

In addition to standardizing seed content by weight, seed content was also estimated on a per fecal unit basis. This approach was necessary given the substantial variation in fecal mass among ungulate species, each of which produces different quantities of dung per defecation (averaged DW per defecation: 49.6 g for deer, 630 g for cow; 300 g for horse, 12.9 g for wild board (see details on variation range and source of information in S1 Table). Thus, herbivore species likely represent a major source of variation in seed deposition via feces, a factor particularly relevant to understanding spatial seed dispersal patterns [31,52]. To account for these differences, the number of seeds potentially deposited at each deposition point was estimated as the product of the standardized seed content (seeds per gram DW) and the average dry weight per defecation for each species, based on values from published sources (S1 Table).

### 2.4. Data analyses

#### 2.4.1. Taxonomic composition of dispersed seeds. Effect of site and of ungulate species.

To evaluate the potential effect of site (i.e., Martinazo *vs*. Matasgordas) on the taxonomic composition of dispersed seeds, a non-metric multidimensional scaling (NMDS) analysis was performed using Euclidean distance as the similarity index. This index was chosen over alternatives such as Bray–Curtis due to the importance of zero values (absence of a seed family) in our dataset, as Bray–Curtis assigns lower relative weight to zeros. Seed content was expressed both per gram DW and per fecal unit. Additionally, a PERMANOVA (Permutational Multivariate Analysis of Variance) was conducted to detect the significance of differences. This analysis included only data from the ungulate species common to both sites (i.e., deer), excluding the two wild boar samples and the outlier sample S72 from Matasgordas, and thus considering only deer samples from Martinazo (representing 48.7% of the total samples from that site). NMDS based on Euclidean distance was also applied to assess potential differences in the taxonomic composition of dispersed seeds among the four ungulate species. PERMANOVA was again used to test for overall significant differences among species, and Bonferroni correction was applied for pairwise comparisons between them. All statistical analyses were conducted using the PAST v4.17 software package.

#### 2.4.2. Spatial pattern of seed dispersal.

Techniques of marked point pattern analysis [53,54] were used to evaluate potential spatial patterns in herbivore-generated seed rain at Martinazo and Matasgordas. All spatial point pattern analyses were conducted with *Programita* software [54] available at http://programita.org/. The approach was applied first to the total number of dispersed seeds and then to those from the most frequently dispersed plant families that were present in a sufficient number of samples to allow for robust analysis. The datasets comprised spatial coordinates (*xi*) and marks (*mi*) representing seed content (i.e., number of seeds). Seed content is expressed per fecal unit to provide a more realistic representation of seed arrival in the presence of different ungulate species. The data structure consists of a univariate quantitatively marked point pattern, where the coordinates (*xi*) represent the univariate point pattern and the seeds content (*mi*) represent a quantitative attribute (i.e., mark).

Mark correlation functions are based on all (ordered) pairs of fecal units whose inter-point distances lie within a small interval (*r* − *h*, *r* + *h*). The parameter *h,* called the bandwidth, must be sufficiently large to provide an adequate number of pairs in each distance class “*r”* but small enough to resolve relevant biological details [53] The objective of mark correlation functions is to estimate the mean value *c*t(*r*) of a test function *t*(*m*_*i*_, *m*_*j*_) for two marks *m*_*i*_ and *m*_*j*_, taken over all (ordered) pairs of fecal units *i* – *j* with inter-point distances of *r* ± *h*.

This procedure is then repeated across a range of distances *r,* using intervals Δ*r* to produce the non-normalised mark correlation function *c*t(*r*) [53]. To obtain the final mark correlation function, *c*t(*r*) is normalized by the expected value *c*t of the test function taken over all pairs of fecal units, regardless of their spatial separation:


$$k_{t}\;(r)\;=\;c_{t}(r)/c_{t}$$ (1)

Many different test functions *t*(*m*_*i*_, *m*_*j*_) are possible. Here we use three of them which are complementary in the information they provide [54,55]. Specifically, we used the *r*-mark correlation function *k*_m_(*r*), which is based on the test function:


$$t(m_{i},\;m_{j})=\;m_{i}$$ (2)

The estimator of the corresponding non-normalised mark correlation function is given by:


ct^(r)=μ(r)=∑i=1n∑j=1,≠nmi×kh(‖xi−xj‖ − r)∑i=1n∑j=1,≠nk(‖xi−xj‖ − r) (3)

Where the “box kernel” function *k*^*h*^*(d)* yields a value of 1/2*h* if the two fecal units with coordinates x*i* and x*j* are at a distances of *r* ± *h*, and zero otherwise [53,54]. Thus, the denominator of Eq. (3) corresponds to the number of ordered pairs of fecal units *i* and *j* that are distances *r* ± *h* apart; Eq. (3) calculates the mean mark *m*_*i*_ of the first fecal units *i* of these pairs. The normalisation constant *c*_t_ of the *r*-mark correlation function, which is taken over all pairs of points regardless of their spatial separation, is obtained by replacing *k*^*h*^(*d*) in Eq. (3) with 1/2*h*:


ct^  =∑i=1n∑j=1,≠k nmi2h∑i=1n∑j=1,≠kn(12h) =1n ∑inmi=μ (4)

and yield μ, the mean value of m_i_ taken over all fecal units *i* (Illian et al. 2008).

Thus, for the r-mark correlation function, *km(r)>1* indicates a positive effect of feces aggregation (i.e., the seed content of feces that have nearby feces at distance *r* is on average larger than the average seed content across feces), while *km*(*r*) < 1 indicates a negative effect (i.e., the seed content of feces that have nearby feces at distance *r* is on average smaller than the average seed content) (e.g., [54]).

We also used a mark correlation function that characterizes the spatial covariance in seed number of two fecal units separated by distance *r*. This test function is known as Schlather’s and was proposed by Schlather, Ribeiro and Diggle [55].


$$t(r,\;m_{i},m_{j})=[m_{i}-\mu \;(r)][m_{j}-\mu (r)]$$ (5)

It results in a Moran’s I-like summary statistic I_mm_ (r), a spatial variant of the classical Pearson correlation coefficient [57]. I_mm_ (r) is normalized by mark variance σ^2^. Thus, I_mm_ (r) is the unequivocal Pearson correlation coefficient between the two variables m_i_ and m_j_ defined by the ordered i-j pairs of feces separated by distance *r* ± h. Note that a test function that adjusts for the mean μ(*r*) that considers only pairs of fecal units separated by a given distance *r*, not the population mean μ, is required to get a summary statistic that can be interpreted as a correlation coefficient [56].

Finally, we use the density correlation function *Cm,K*(*r*) developed by Fedriani et al. [55], which directly relates the seed content in a fecal unit to the density of its nearby feces. *C*_*m,K*_(*r*) estimates the classical Pearson correlation between the seed content *m*_*i*_ of a fecal unit and the number of nearby feces within distance *r* [=λ*K*_*i*_(*r*)]. Thus, the density correlation function is based on the following test function:


$$t(r,m_{i},K_{i})=[m_{i}-\mu ][\lambda K_{i}(r)-\lambda K(r)],$$ (6)

where *m*_*i*_ is the seed content of focal fecal unit *i*, μ is the mean seed content of the feces population, λ is the overall density of feces in the study area, λ*K*_*i*_(*r*) is the number of nearby feces around focal fecal unit *i* within distance *r* and λ*K*(*r*) is the mean number of nearby feces within distance *r* for all fecal units (see Supporting Information S1-S3).

## 3. Results

### 3.1. Vegetation physiognomy at the seed arrival microsites, and importance of different dispersers at each site

As expected, vegetation physiognomy at the seed arrival microsites in both Martinazo and Matasgordas was predominantly composed of open grasslands. Thus, 78–82% microsites were exclusively covered by grassland (Table 1), while 18–22% microsites were partially occupied by shrubs (= shrubby microsites) with moderate (28–31%) cover. *Halimium halimifolium* was the dominant shrub species in most shrubby microsites at both locations, although the second most frequently dominant species differed: *Stauracanthus genistoides* in Matasgordas and *Asparagus acutus* in Martinazo. These assessments are not based on statistical comparisons. Though a methodology specifically designed for vegetation analysis would be more appropriate for a comprehensive assessment, it is beyond the scope of this study.

**Table 1:** Vegetation physiognomy in seed arrival microsites at the two study sites.

Sites	Martinazo	Matasgordas
% Open microsites (covered only by grasslands)	82	78
% Microsites with shrub presence (=shrubby microsites)	18	22
Averaged shrub cover (in %) in the shrubby microsites	28	31
Dominant shrub species in shrubby microsites (% microsites dominated by a species)		
*Halimium halimifolium*	63	59
*Stauracanthus genistoides*	12	38
*Asparagus acutus*	25	3

As anticipated, only wild ungulates feces were found in Matasgordas (Table 2), with deer strongly dominant (98.3% feces) and wild boar negligible (2 feces; 1.7% of total). In Martinazo, deer were also most abundant (49% feces), followed by cattle (27% feces) and horses (12% feces) and wild boar (12% feces) in much lower proportion. Overall, fecal density was considerably lower in Matasgordas (1.3 feces.ha^-1^) than in Martinazo (11.4 feces.ha^-1^).

**Table 2:** Seed content (expressed per gram dry weight and per fecal unit; DW) in deposits from different ungulates at Martinazo and Matasgordas (mean values ± se). The seed content per fecal unit is calculated from average feces DW of different ungulates (S1 Table). Cumulated number of dispersed seeds is the sum of seeds in all deposits from each ungulate.

	Deer	Cow	Horse	Wild boar	All ungulates pooled
**Martinazo**					
*Average seed content*					
Standardized (seeds.g^-1^)	6.0 (±0.8)	5.6 (±1.6)	4.9 (±1.5)	5.3 (±1.7)	5.7 (±0.6)
Per fecal unit (seeds.deposition^-1^)	300 (±42.3)	3,514 (±1021)	1,480 (±451)	69 (±22)	1,302 (±315)
*Cumulated number of seeds dispersed*					
Total seeds per ungulate species	16,514	108,941	20,724	903	147,082
% Dispersed seeds per ungulate species	11.2	74.1	14.1	0.6	100
**Matasgordas** ^**1**^					
*Average seed content*					
Standardized (seeds.g^-1^)	12.3 (±0.5)	–	–	16.8 (±10.3)	12.4 (±1.4)
Per fecal unit (seeds.deposition^-1^)	615 (±24)	–	–	218 (±134)	606 (±69.6)
*Cumulated number of seeds dispersed*					
Total seeds per ungulate species	71,349	–	–	437	71,786
% Dispersed seeds per ungulate species	99.4	–	–	0.6	100

^1^Values in Matasgordas site exclude the outlier sample S72. Including sample S72 in the analyses would result in a standardized seed content of 23 ± 83 seeds.g^-1^ in deer feces from Matasgordas, corresponding to 1,150 ± 4,150 seeds per deposition and total of 135,581 seeds dispersed by deer.

Overall seed content varied from 0 to 97 seeds.g^-1^ across all samples, excluding the outlier S72 (Table 2). Seed content per gram (DW) was generally higher in Matasgordas than in Martinazo (Table 2), but the opposite pattern was observed when expressed per fecal unit. This reflects the nearly exclusive presence of deer in Matasgordas which produce lighter droppings with lower seed content than cattle and horses (S1 Table), species that, alongside deer and wild boar, are present in Martinazo.

### 3.2. Taxonomic composition of dispersed seeds. Effect of site and ungulate species

Seeds extracted from fecal samples were predominantly small (<1mm), rounded, and hard-coated. Of the total 7,074 seeds recovered, only 233 (3.3%) could not be identified, while the rest were identified to various taxonomic levels. In total, 21 families, 53 genera, and 57 species were identified (S2 Table). Family was determined for the majority (96.7%) of seeds and was therefore selected for subsequent analysis of taxonomic composition.

**Fig 2:**
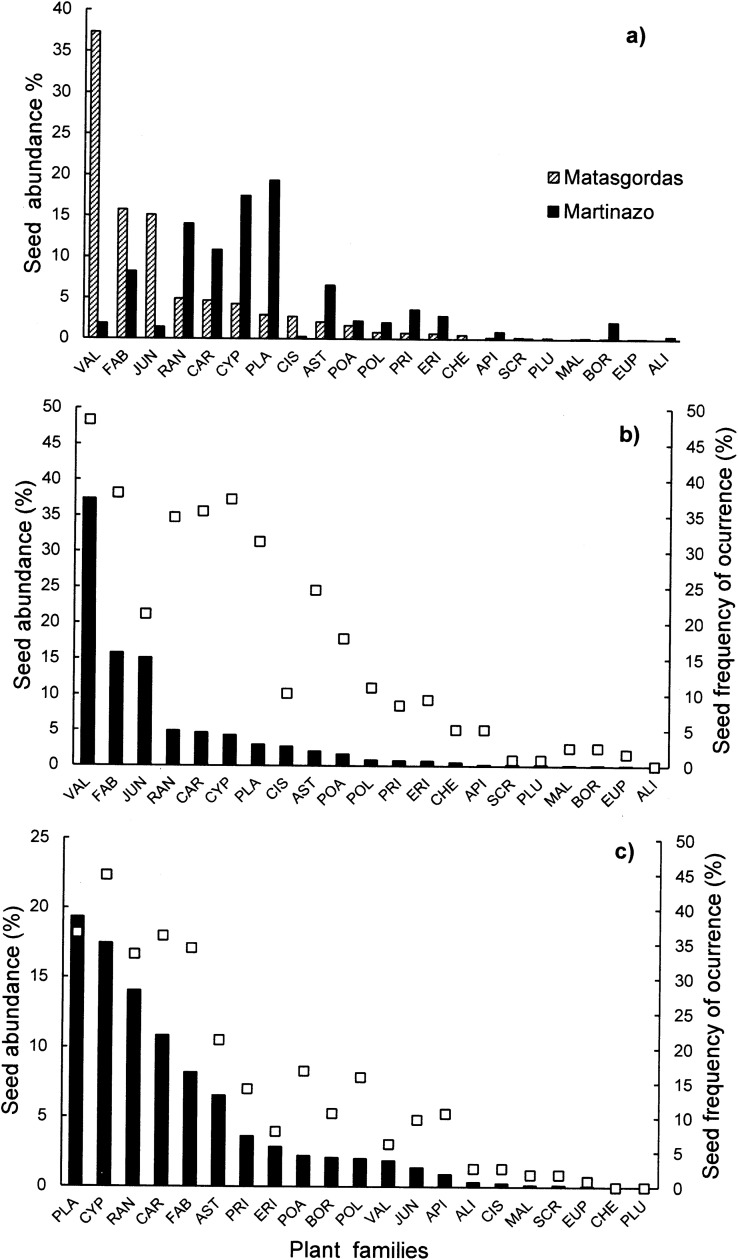
(a) Relative abundance of different plant families (percentage of the seed pool) at the studied sites, and combined relative abundance of plan families (= black bars) and their frequency (percentage of samples where a family occurs = white squares) in Matasgordas (b) and Martinazo (c). The sample S72 is excluded from computation. Plant families: VAL(*Valerianaceae*), FAB (*Fabaceae*), CYP (*Cyperaceae*), PLA (*Plantaginaceae*), RAN (*Ranunculaceae*), CAR (*Caryophyllaceae*), AST (*Asteraceae*), CIS (*Cistaceae*), POA (*Poaceae*), PRI (*Primulaceae*), ERI (*Ericaceae*), POL (*Polygonaceae*), BOR (*Borraginaceae*), API (*Apiaceae*), CHE (*Chenopodiaceae*), SCR (*Scrophulariaceae*), MAL (*Malvaceae*), ALI (*Alismataceae*), PLU (*Plumbaginaceae*), EUP (*Euphorbiaceae*).

The importance (i.e., relative abundance and frequency of occurrence) of different plant families in the seeds found in Matasgordas and Martinazo fecal samples (excluding S72) is shown in Fig 2. The large majority of dispersed plant families were present at both sites (i.e., 17 families, Fig 2a) while three were exclusive to Matasgordas (i.e*., Chenopodiaceae, Plumbaginaceae, Euphorbiaceae*) and one exclusive to Martinazo (*Alismataceae*). Exclusive families were scarce at their respective sites with abundances ranging from only 0.07 to 0.49%. For the most abundant families, the abundance ranking varied between the two sites. The most abundant dispersed family in Matasgordas (*Valerianaceae,* 37%; Fig 2a) was very scarce in Martinazo (1.9%). This family was represented by a single species (*Centranthus calcitrapae*; S2 Table). Similarly, the second and third most abundant families in Matasgordas (*Fabaceae* [16%] and *Juncaceae* [15%]) were moderately abundant or scarce in Martinazo (*Fabaceae* [8.2%] and *Juncaceae* [1.4%]). The *Juncaceae* family was represented by a single genus, *Juncus* (S2 Table) which was also the only genus present in the outlier sample S72. Conversely, the three most abundant dispersed families in Martinazo (*Plantaginaceae,* 19%; *Cyperaceae,* 17%; *Ranunculae*, 14%) were scarce in Matasgordas (*Plantaginaceae*, 3%; *Cyperaceae,* 4%; *Ranunculae,* 5%). It is also noteworthy that seed abundance was fairly unevenly distributed across families in Matasgordas (Fig 2b), where the most abundant family *(Valerianaceae)* was considerably more abundant than the next two (*Fabaceae* and *Juncaceae).* In contrast, seed abundance in Martinazo was more evenly distributed (Fig 2c) with the three most abundant families (i. e., *Plantaginaceae, Cyperaceae* and *Ranunculaceae)* occurring in similar proportions.

The discrepancy between the frequency of occurrence and relative abundance of dispersed families was particularly pronounced at Matasgordas (Fig 2b), where several families exhibited disproportionately high frequency relative to their relative abundance (e.g., *Fabaceae, Ranunculaceae, Caryophyllaceae, Cyperaceae, Plantaginaceae, Asteraceae, Poaceae*). At Martinazo (Fig 2c), only a few families (namely *Fabaceae* and *Caryophyllaceae*) showed marked discrepancies. Notably, *Fabaceae* and *Caryophyllaceae* consistently exhibited high discrepancies at both sites, being frequently dispersed despite their low relative abundance. This suggests that dispersers frequently consume seeds from these families, albeit in small quantities.

**Fig 3:**
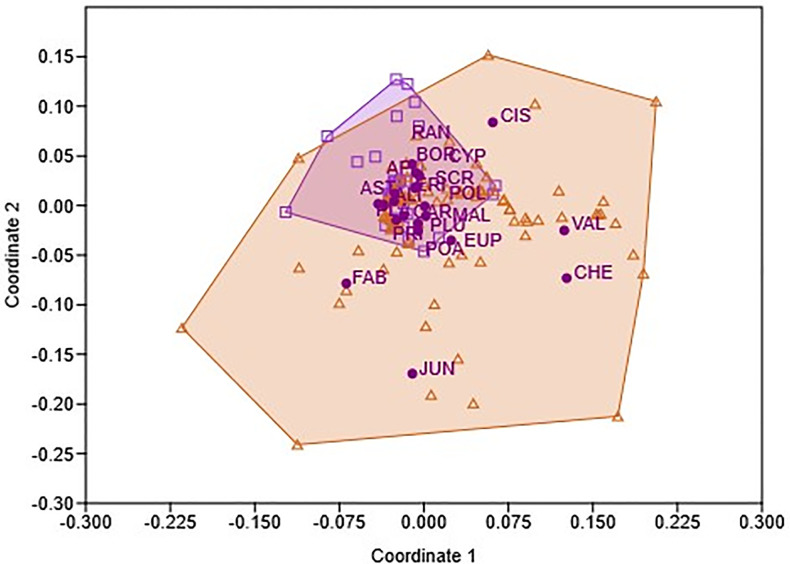
NMDS results showing deer samples from Martinazo and Matasgordas sites The analysis is based on seeds per gram DW. Squares and purple area = Martinazo deer, triangles and pink area = Matasgordas deer. Initials next to dots are seed families positioned by abundance and frequency in each site. Initials of family names as in Fig 2.

NMDS results (Fig 3) comparing the taxonomic composition of seeds dispersed by deer at Matasgordas (n = 117) and Martinazo (n = 56) reveal substantial overlap across families. Nonetheless, several seed families were exclusive or more prominently represented in Matasgordas (e.g., *Valerianaceae, Juncaceae, Fabaceae, Chenopodiaceae and Cistaceae),* others were better represented in Martinazo (e.g., *Ranunculaceae* and *Borraginaceae*). The stress value (0.20) provides a fair but not strong representation of community dissimilarities. Coordinate 1 and Coordinate 2 explained relatively low proportions of the variation (R² = 0.10 and 0.17 respectively), which indicate that the taxonomic differences between the two sites in terms of deer seed dispersal were relatively moderate. However, PERMANOVA analysis indicated significant differences between the two sites (Df = 1, F = 7.01, *P* = 0.0001). In this analysis, seed content was expressed per gram of dry weight. Nonetheless, when expressed per fecal unit, results remained consistent (data expressed per fecal unit available at https://doi.org/10.12795/11441/176810).

**Fig 4:**
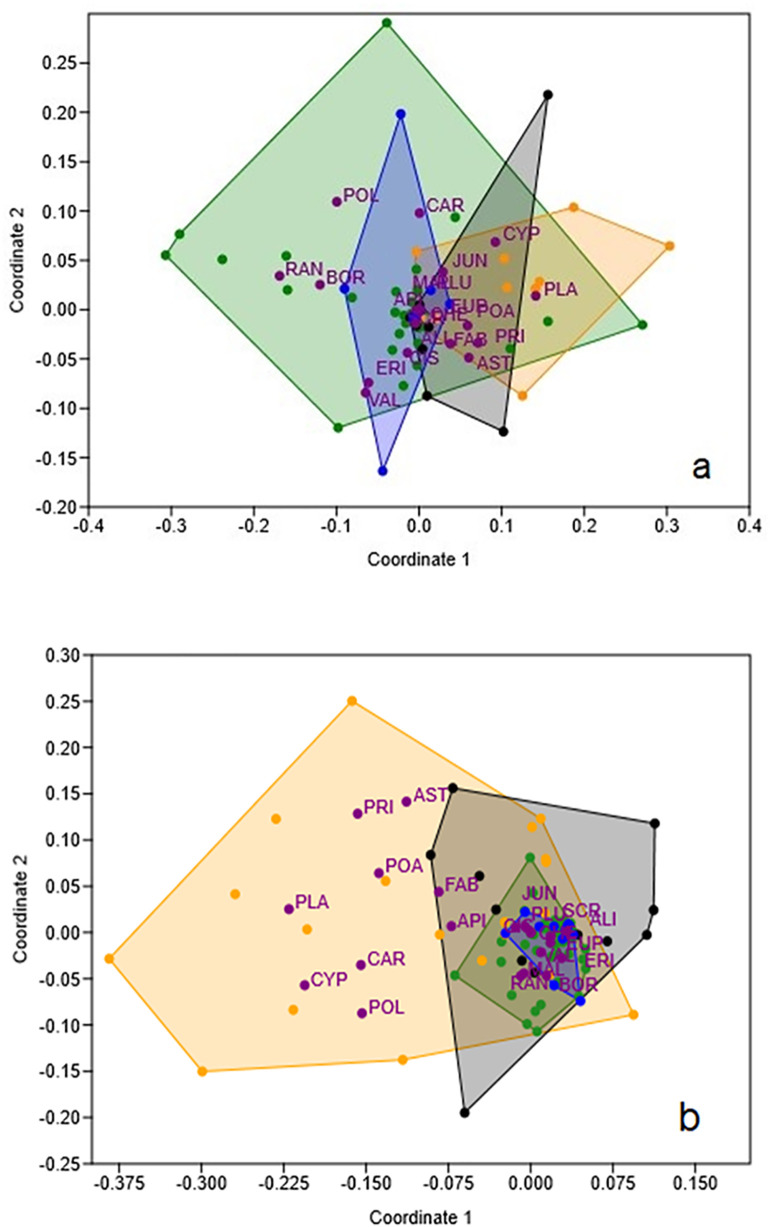
NMDS results showing samples of different ungulate species (dots and areas of different colors) at Martinazo: Green = deer; yellow = cow; black = horse; blue = wild boar. Seed content expressed per gram DW **(a)** and per fecal unit (b). Initials next to dots are families of seeds positioned by abundance and frequency in each ungulate. Initials of family names as in Fig 2.

NMDS comparing taxonomic composition of seeds dispersed by different ungulates at Martinazo (n = 57, 31, 14 and 13 samples for deer, cow, horse and wild boar respectively) is shown in Fig 4. As above, there was evident overlap in seed composition when data were expressed per gram DW (Fig 4a). Nevertheless, certain plant families were preferentially associated with specific seed dispersers. Deer and cattle were the most dissimilar ungulates in the taxonomic composition of dispersed seeds, with *Ranunculaceae*, *Polygonaceae and Borraginaceae* families more closely associated with deer, and *Plantaginaceae* with cattle. Wild boar and horse occupied an intermediate position: wild boar showed strong overlap with deer and partial overlap with cow and horse, while horse overlapped closely with both deer and cow. Stress value in the NMDS ordination was low (0.10) indicating a good fit between ordination and original dissimilarities. Coordinate 1 (R² = 0.72) and Coordinate 2 (R² = 0.48) captured the dominant gradient in seed composition with separation among the ungulate species. PERMANOVA analysis (Table 3, column A) also revealed significant overall differences among ungulate species (Df = 3, F = 2.142, P = 0.012). However, pairwise comparisons using the Bonferroni-corrected test indicated that only the deer–cow comparison was statistically significant.

**Table 3:** Permanova results for general differences in the composition by families of dispersed seeds by different ungulate species in Martinazo site. Analyses were conducted on seed content expressed both, as seeds per gram DW (column A) and as seed per fecal unit (column B). Significant differences after Bonferroni corrected pairwise comparisons for the four ungulates are shown in bold font.

	Seed content			
	A) Per unit DW (seeds.g^1^)		B) Per fecal unit (seeds.deposition^-1^)	
Permanova	Df = 3, F = 2.142; *P* = 0.012		DF = 3, F = 6.61; *P* = 0.0003	
Pairwise comparisons	F	*P*	F	*P*
Deer – Cow	**3.619**	**0.043**	**14.4**	**0.0006**
Deer – Horse	2.137	0.345	**9.88**	**0.0006**
Deer – Wild boar	0.774	0.998	1.534	0.9138
Cow – Horse	2.086	0.573	2.374	0.5976
Cow – Wild boar	1.861	0.7596	3.531	0.1806
Horse -Wild boar	1.441	0.457	**2.852**	**0.0114**

Similar results were obtained when NMDS analysis was conducted using data expressed per fecal unit, although in this case a greater point dispersion was observed along Coordinates 1 and 2 (Fig 4b). As in previous analysis (Fig 4a), the *Plantaginaceae* family remained closely associated with cow. However, in this case several other families also showed strong associations with cow, including *Cyperaceae, Primulaceae, Poaceae, Cariophyllaceae* and *Polygonaceae*. Consistent with previous results, the *Ranunculaceae* family was again associated with deer, and additionally, the *Juncaceae* family showed a similar association in this analysis. *Fabaceae* and *Apiaceae* were primarily associated with horses, while no plant family was differentially associated with wild boar. The NMDS ordination yielded a moderate stress value (0.18), indicating a fair representation of dissimilarities. Coordinate 1 (R² = 0.85) represented the dominant compositional gradient separating the major ungulate groups, whereas Coordinate 2 (R² = 0.26) explained a secondary gradient. PERMANOVA results (Table 3, column B) also revealed significant overall differences in the taxonomic composition of dispersed seeds among ungulate species, with a higher level of significance than in the previous analysis (Df = 3, F = 6.61, P = 0.0003). Furthermore, a greater number of significant pairwise differences were detected, specifically between deer and cattle, deer and horse, and boar and horse.

### 3.3. Spatial patterns of seed dispersal

**Fig 5:**
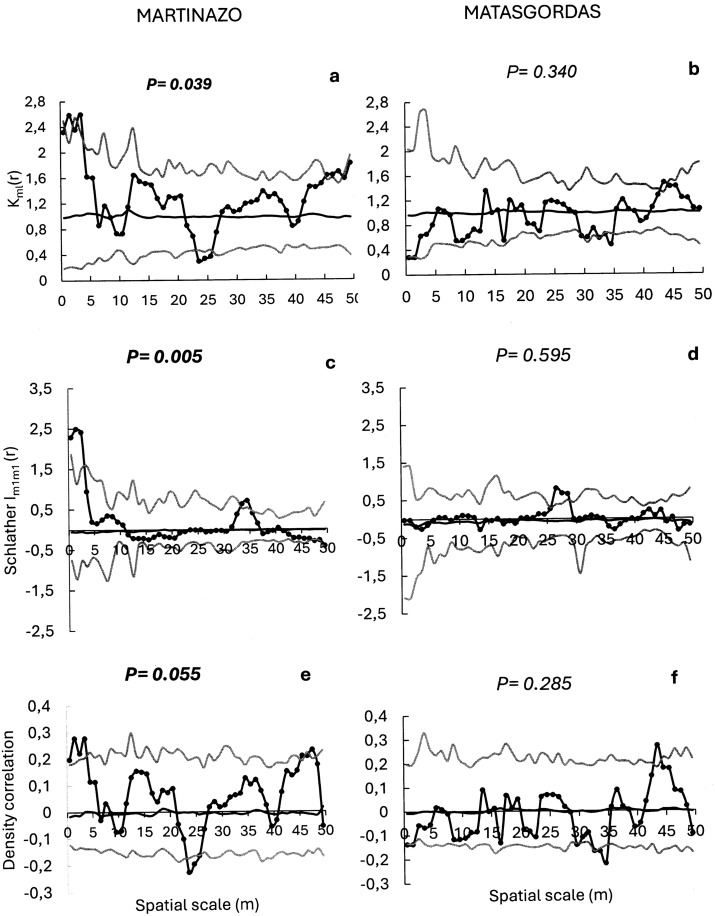
Mark correlation functions to evaluate potential spatial structure in total seed number dispersed by ungulates in Martasgordas (b,d,f) and Martinazo (a,c,e). The r-mark correlation function (a, b) describes the mean number of seeds (m_i_) in a fecal unit at distance “r” of another unit. Schlather’s correlation function (c, d) quantifies the correlation between the number of seeds in two different units separated by distance “r”. Density correlation function (e, f) assesses the correlation between the number of seeds and the density of fecal units distance “r”. Observed functions = solid dots. Expected functions under the null model of random seed content distribution = black line. Simulation envelopes = grey lines (fifth lowest and fifth highest values of the functions created by 199 simulations under random labelling). Goodness-of-fit [58] is used to test the overall fit of the random marking null model for the entire distance up to 50 m. Significant *p*-value from GoF test in bold font indicates significant departures of the observed function from the random null model.

Spatial pattern of seeds dispersal (i.e., total number of seeds per fecal unit) produced different outcomes at the two sites under study (Fig 5). At Martinazo, a significant r-mark correlation function (P = 0.039, Fig 5a) indicates that seed content was influenced by the distance between fecal deposits. A positive effect of feces aggregation was observed at short distance (r = 0.5 to 2.5m), where the observed r-mark correlation function exceeded the upper envelope, while a negative effect was apparent at intermediate distances (r = 23.5 to 26.5 m), where it fell below the lower envelope. These results suggest a complex dependence of seed content on the spacing of fecal deposits. In Matasgordas, the observed r-mark correlation function fell within the simulation envelope across all scales, indicating no relationship between seeds content and the distance between fecal deposits (P = 0.340, Fig 5b). Schlather’s correlation function which assesses spatial covariance in seed content between two fecal deposits separated by a distance r, was also significant in Martinazo (P = 0.005, Fig 5c). The function notably exceeded the upper envelope at short distances (r = 0.5 to 2.5 m) indicating that the nearby fecal deposits had higher than expected similarity in seed content. In addition, the Schlather’s correlation function slightly exceeded the upper envelope at greater distances (r = 33.5 to 34.5 m). At Matasgordas, this function was non-significant (P = 0.595, Fig 5c), indicating no spatial covariance or similarity in seed content among fecal deposits at any scale. The density correlation function which relates seed content in fecal deposits to nearby deposit density was marginally significant at Martinazo (P = 0.055, Fig 5e), exceeding the upper envelope at short (r = 0.5–2.5 m) and long (r = 47 m) distances. At Matasgordas, this function was non-significant (P = 0.285, Fig 5f).

**Fig 6:**
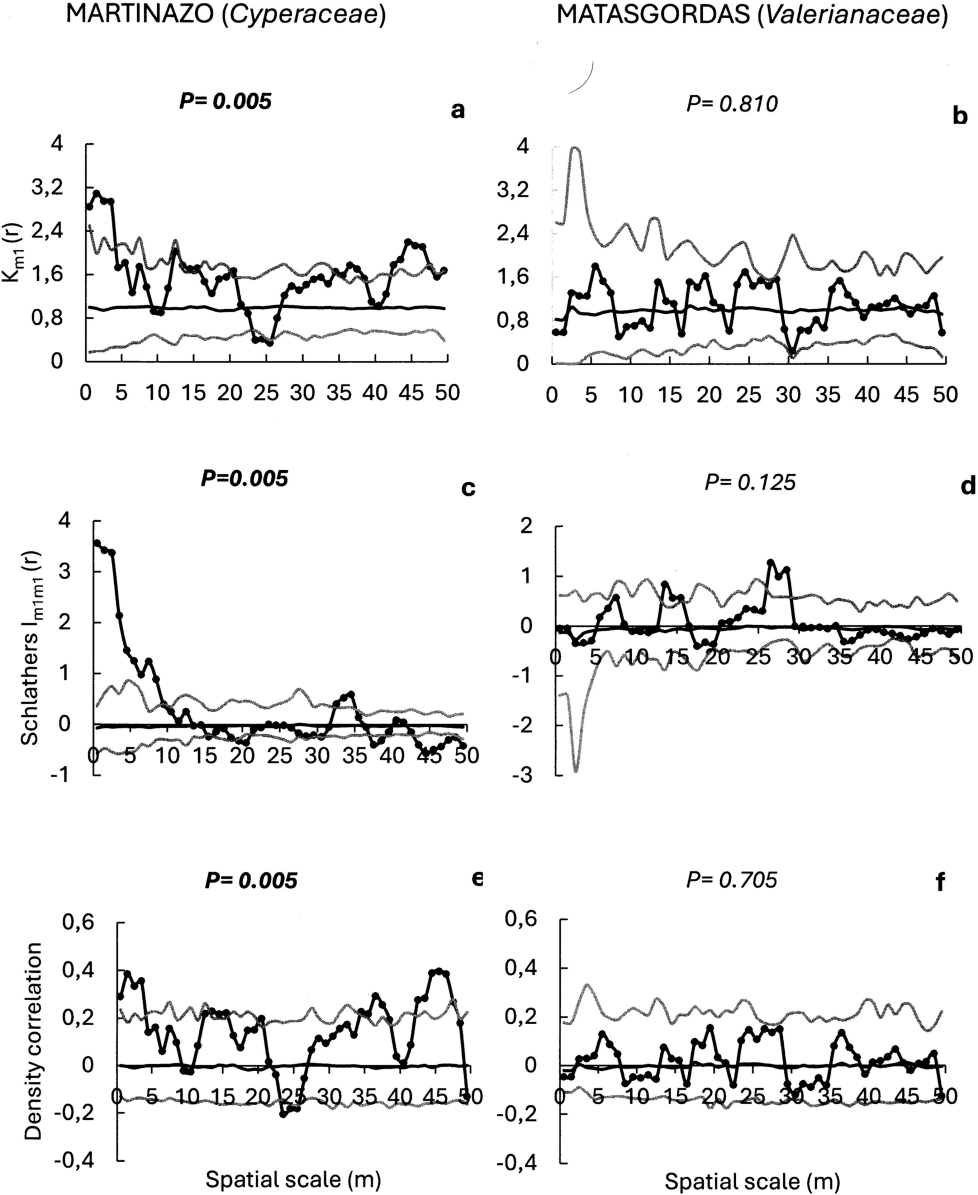
Mark correlation functions to evaluate potential spatial structure in the seeds number of frequent plant families (in bracket) in Martasgordas (b, d, f) and Martinazo (a, c, e). The r-mark correlation function (a,b) describes the mean number of seeds (mi) in a fecal unit at distance “r” of another fecal unit. Schlather’s correlation function (c, d) quantifies the correlation between the number of seeds in two different fecal units separated by distance “r”. Density correlation function (e, f) assesses the correlation between the number of seeds and the number of nearby fecal units at a distance “r”. Observed functions = solid dots. Expected functions under the null model of random seed content distribution = black line. Simulation envelopes = grey lines (fifth lowest and fifth highest values of the functions created by 199 simulations under random labelling). Goodness-of-fit [58] is used to test the overall fit of the random marking null model for the entire distance up to 50 m. Significant *p*-value from GoF test in bold font indicates significant departures of the observed function from the random null model.

**Fig 7:**
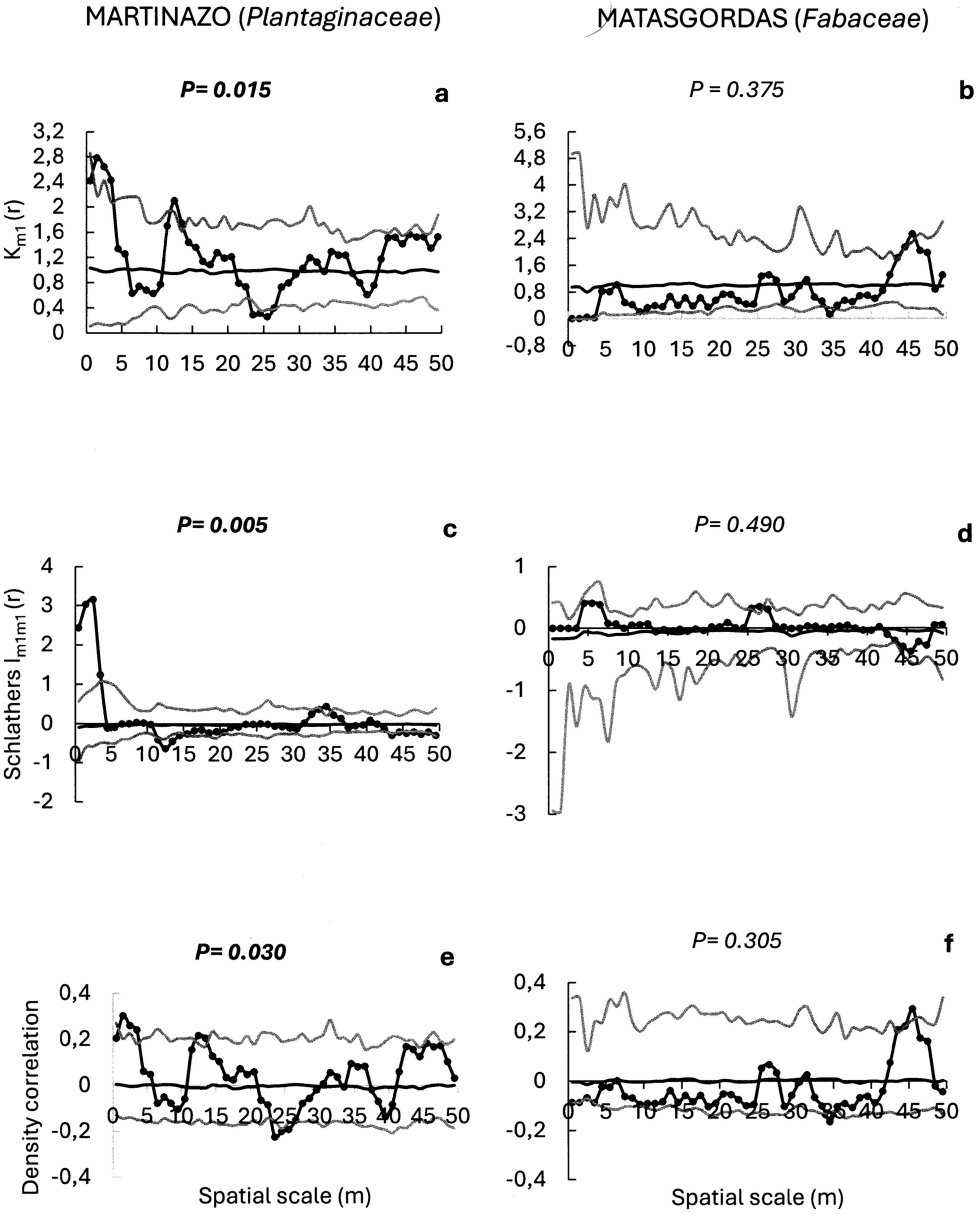
Mark correlation functions to evaluate potential spatial structure in the seed number of frequent plant families (in brackets) in Martasgordas (b,d,f) and Martinazo (a,c,e). The r-mark correlation function (a,b) describes the mean number of seeds (mi) in a fecal unit at distance “r” of another fecal unit. Schlather’s correlation function (c, d) quantifies the correlation between the number of seeds in two different fecal units separated by distance “r”. Density correlation function (e, f) assesses the correlation between the number of seeds and the number of nearby fecal units at a distance “r”. Observed functions = solid dots. Expected functions under the null model of random seed content distribution = black line. Simulation envelopes = grey lines (fifth lowest and fifth highest values of the functions created by 199 simulations under random labelling). Goodness-of-fit [58] is used to test the overall fit of the random marking null model for the entire distance up to 50 m. Significant The *p*-value from GoF test in bold font indicates significant departures of the observed function from the random null model.

**Fig 8:**
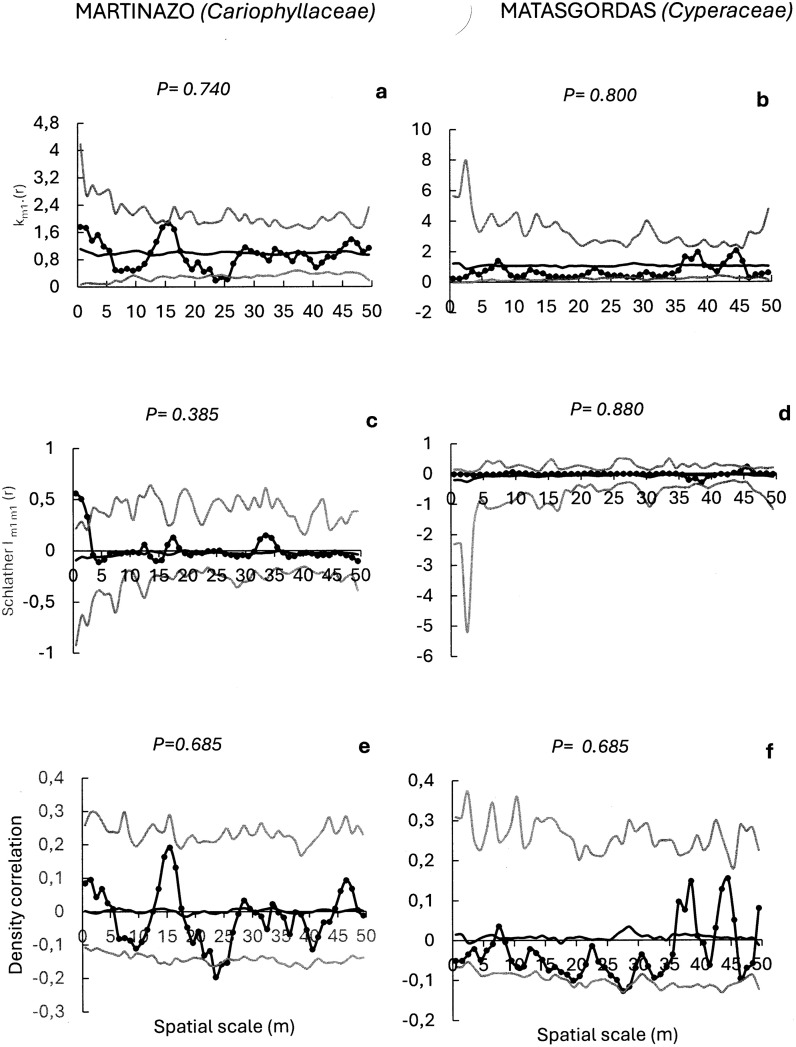
Mark correlation functions to evaluate potential spatial structure in the seed number of frequent plant families (in brackets) in Martasgordas (b,d,f) and Martinazo (a,c,e). The r-mark correlation function (a,b) describes the mean number of seeds (mi) in a fecal unit at distance “r” of another fecal unit. Schlather’s correlation function (c, d) quantifies the correlation between the number of seeds in two different fecal units separated by distance “r”. Density correlation function (e, f) assesses the correlation between the number of seeds and the number of nearby fecal units at a distance “r”. Observed functions = solid dots. Expected functions under the null model of random seed content distribution = black line. Simulation envelopes = grey lines (fifth lowest and fifth highest values of the functions created by 199 simulations under random labelling). Goodness-of-fit [58] is used to test the overall fit of the random marking null model for the entire distance up to 50 m. Significant The p-value from GoF test in bold font indicates significant departures of the observed function from the random null model.

With regard to the plant families most frequently dispersed by ungulates at the two sites, we evaluated the potential spatial structure of seed dispersal for *Cyperaceae*, *Plantaginaceae* and *Cariophyllaceae* seeds in Martinazo (with frequencies of occurrence of 44.7%, 36.3%, and 36.0%, respectively), and for *Valerianaceae*, *Fabaceae* and *Cyperaceae* seeds in Matasgordas (with frequencies of occurrence of 48.3%, 38.1%, and 37.3%, respectively) (Figs 6–8).

The r-mark correlation function for the *Cyperaceae* seeds in Martinazo, was significant (P = 0.005, Fig 6a) indicating that the number of seeds per fecal unit was influenced by the distance between fecal deposits. As above (Fig 5a), there was a positive effect of feces aggregation on seed content (km (r) >1) at short distance (r = 0.5 to 3.5 m) where the observed function exceeded the upper envelope. Additionally, the function surpassed the upper envelope at longer distances (r = 42.5–46.5 m). The *Valerianaceae* seeds in Matasgordas, did not show a relationship between seed content and the distance between nearby fecal units (P = 0.81 Fig 6b). Schalater’s correlation function was significant for *Cyperaceae* seeds in Martinazo (P = 0.005, Fig 6c), where the observed function greatly exceeded the upper envelope at short distance (r = 0.5 to 8.5m) and moderately at intermediate distance (r = 32.5 to 34.5 m). Conversely, this function was not significant for *Valerianaceae* in Matasgordas (P = 0.125; Fig 6d). Finally, density correlation function, was significant for *Cyperaceae* in Martinazo (P = 0.005; Fig 6e) exceeding the upper envelope at short (r = 0.5 to 3.5 m) and long distances (r = 36.5 to 37.5 and 42.5 to 46.5 m). These results indicated a complex spatial dependence between seed content and nearby feces density. In contrast, this function was not significant for *Valerianaceae* in Matasgordas (P = 0.705; Fig 6f) with the observed function falling within the simulation envelope at all scales.

With respect to the *Plantaginaceae* family in Martinazo, a significant r-mark correlation function (P = 0.015, Fig 7a) indicated a spatial dependence between seed content and nearby feces distance. The positive effect of feces aggregation (indicated by km(r) > 1) for *Plantaginaceae* in Martinazo was strongest at short distances (r = 1.5–3.5 m), where the observed function exceeded the simulation envelop. In contrast, the *Fabaceae* seeds in Matasgordas did not show significant r-mark correlation function (P = 0.375; Fig 7b), indicating no relation between seed content and distance between nearby fecal units at any scale. The Schlather’s correlation function for *Plantaginaceae* seeds in Martinazo, also indicates significant covariance in seed content of nearby fecal units (P = 0.03, Fig 7c), and again the observed function greatly exceeds the envelope at short distance (r = 0.5 to 3.5 m). Conversely the non-significant Schlather’s correlation function for *Fabaceae* seeds in Matasgordas (P = 0.490; Fig 7d) indicates no relationship between number of dispersed seeds and the distance between feces. Finally, a significant density correlation function for *Plantaginaceae* seeds in Martinazo (P = 0.03; Fig 7e) suggests dependence between seeds content and nearby feces density. The density correlation function exceeded the upper simulation envelope at short distances (r = 1.5 to 3.5 m) and fell slightly below the lower envelope at intermediate distance (r = 23.5 to 25.5 m), reflecting again a complex spatial relationship. In contrast, the non-significant density correlation function for *Fabaceae* seeds in Matasgordas (P = 0.305; Fig 7f) indicates no dependence between seed content and nearby feces density.

Finally, the non-significant r-mark correlation function for dispersed *Cariophyllaceae* seeds in Martinazo (P = 0.740, Fig 8a) and for *Cyperaceae* seeds in Matasgordas (P = 0.80; Fig 8b) indicated that seed content in the fecal units was not influenced by the distance to nearby fecal units in either case. Similarly, non-significant Schlather’s correlation function for dispersed *Cariophyllaceae* in Martinazo (P = 0.385, Fig 8c) and for dispersed *Cyperaceae* seeds in Matasgorgas (P = 0.880, Fig 8d) show no spatial covariance in seeds content at any scale. Furthermore, density correlation function for *Caryophillaceae* in Martinazo and for *Cyperaceae* in Matasgordas were also not significant (P = 0.685; Fig 8e and P = 0.685; Fig 8f respectively) indicating that seed content in fecal units was not influenced by nearby feces density in either case.

## 4. Discussion

### 4.1. Effect of site on seed dispersal

Previous studies have reported contrasting patterns of seed dispersal by sympatric ungulates. While some suggest significant similarities [7,59] others highlight clear interspecific differences among ungulates [11,12]. Our results reveal quantitative differences in herbaceous seed dispersal among grasslands sites in Doñana National Park. Seed deposition per gram was more than twice as high at Matasgordas (12.4 seeds·g ⁻ ¹) compared to Martinazo (5.7 seeds·g ⁻ ¹; Table 2). However, when assessed per fecal unit, Martinazo showed higher seed loads (1,302 *vs*. 606 seeds.deposition ⁻ ¹), likely due to differences in ungulate assemblages. Martinazo hosts large-bodied domestic ungulates (cattle and horses) with larger fecal deposits and higher seed loads (3,514 and 1,480 seeds.unit ⁻ ¹, respectively), whereas our Matasgordas study plot was mainly used by deer, which disperse fewer seeds per deposition (300–615 seeds.unit ⁻ ¹; Table 2). Nevertheless, the outlier sample S72, excluded from the analysis, was recorded at the Matasgordas site, thereby revealing that highly infrequent events may play an important role in patch formation in these grasslands. These findings underscore the influence of local ungulate composition on seed dispersal quantity [19]. However, it is important to recognize also the likely influence on seed dispersal quantity of the density and composition of the local herbaceous community. Indeed, the Martinazo soil contains more than twice the organic matter, phosphorus and nitrogen of Matasgordas [26] potentially supporting higher herbage and total seed production. Interestingly, however, our results showing the seed load in deer feces from the Matasgordas doubled that found in Martinazo. In Matasgordas deer monopolize the available forage compared to Martinazo, where they share the forage with abundant domestic ungulates. Thus, such unexpected result likely reveal a density-dependent process in food intake and the associated herbaceous seed dispersal [46].

In addition to quantity, seed taxonomic composition differed between sites. Dominant seed families found in ungulate fecal samples at Matasgordas (*Valerianaceae*, *Fabaceae*, *Juncaceae*; Fig 2a) were rare in samples from Martinazo, where *Plantaginaceae*, *Cyperaceae*, and *Ranunculaceae* predominated. These differences likely reflect both variation between study sites in ungulate assemblages and inherent herbaceous plant community heterogeneity, despite similar vegetation physiognomy of the seed arrival microsites (Table 1). Prior studies emphasize that seed content in fecal deposits is shaped by the local plant community [11,18]. Though this study does not isolate ecological or anthropogenic drivers, our analyses (Fig 3) show significant taxonomic differences in deer-dispersed seeds between sites. Certain families were more common in Matasgordas, while others were more frequent in Martinazo, being such variation expected, given the heterogeneous nature of “la vera” grasslands in Doñana [42] and the associated changes in ungulate density and composition [60].

### 4.2. Ungulate species effect on seed dispersal

Our findings highlight the role of ungulate identity in shaping endozoochorous seed dispersal [31]. Significant differences in dispersed seed families were observed among the four ungulate species at Martinazo (Table 3), independent of whether data were expressed per fecal unit or per gram of material. Cattle and deer, in particular, showed clear contrasts: *Plantaginaceae* was strongly associated with cattle, while *Ranunculaceae* was more frequently dispersed by deer (Fig 4). Other associations included *Cyperaceae*, *Primulaceae*, and *Poaceae* with cattle, and *Polygonaceae* and *Boraginaceae* with deer, although these varied depending on the seed content measurement used (Fig 4a, 4b). Additional differences in dispersed seed families emerged between ungulate pairs with markedly different body sizes, specifically between horses and deer, and between horses and wild boar, when data were expressed per fecal unit (Table 3B). For example, differences in digestive physiology and body size among herbivores likely contribute to dietary divergence and resulting seed composition [61,62]. These results are consistent with prior studies showing limited overlap among sympatric ungulates [19] and highlight the importance of body size and behavior in the ecological roles of ungulates [68,69].

Whereas most previous research links differences in seed dispersal to broad habitat preferences [6,11,15], our findings suggest that such differences can also occur at fine spatial scales within a single habitat, the grassland under study. However, relatively small plot sizes and potential long-distance seed transport from adjacent habitats should be considered as they could either increase or decrease detected differences in seed dispersal between sites.

Selective ingestion also appears to play a role. Certain seed families, such as *Fabaceae* and *Caryophyllaceae*, occurred frequently despite low abundance in fecal deposits, suggesting animal selectivity. This pattern was consistent across both sites. *Fabaceae* are known for their high nitrogen content, making them a preferred forage [63–65], and are frequently dispersed by ungulates and other herbivores, while *Caryophyllaceae* (along with *Fabaceae* and *Brassicaceae*) has been identified among the families most frequently dispersed by herbivorous mammals in Mediterranean woodlands [18]. Interestingly, more families exhibited this frequency-abundance mismatch in Matasgordas than in Martinazo, suggesting that factors such as grazers richness may play a role in diet selection, as it has been observed in other studies on free-ranging wild and domestic ungulates [66,67]. However, as mentioned above, other ecological variables may also be involved in the observed differences in plant family’s selectivity, as inferred from frequency-abundance mismatches. These findings underscore the need for further investigation into mechanisms of plant selection and their consequences for seed dispersal by grassland ungulates.

### 4.3. Spatial pattern of seed dispersal

Seed dispersal spatial patterns by ungulates differed markedly between sites. In Matasgordas plot, where deer were virtually the only ungulates, no significant spatial structure was detected in either for total seed content or key seed families (*Valerianaceae*, *Fabaceae*, *Cyperaceae*), indicating an absence of distance-dependent seed distribution. In contrast, Martinazo plot, with a more diverse ungulate community, exhibited clear spatial patterns. Short-distance aggregation of fecal deposits (0.5–2.5 m) was positively associated with increased total seed content, as well as higher counts of *Plantaginaceae* and *Cyperaceae* seeds. These variables (total seeds, *Plantaginaceae* seeds, and *Cyperaceae* seeds) were also positively correlated with the density of fecal deposits at short distance (0.5 to 3.5 m). A positive effect of feces aggregation and feces density was also observed at greater distance (43–46 m) for *Cyperaceae* seeds. In addition, lowest seed content than expected was observed punctually at intermediate distance (23–26 m). Thus, collectively, our mark correlation analyses consistently point to a positive short-range effect of fecal aggregation on total seed input and on *Cyperaceae* and *Plantaginaceae*. “La vera” grasslands in Doñana constitute heterogeneous environments subject to episodic and irregular surface flooding, specially where the groundwater table is shallower as it occurs at Martinazo [26,42]. This hydrological variability may influence local herbaceous communities, ungulate movements, their use of the pasture, and likely contributes to the complex spatial pattern detected.

The short distance positive effect of feces aggregation on seed content at Martinazo plot likely stems from the presence of cattle, whose deposits are large, spatially clumped [20] and have the highest seed content among all the ungulates present in the studied grasslands (Table 2). Studies on mixed ungulate communities in other Mediterranean grasslands have likewise identified cattle as the species dispersing the greatest number of seeds [20]. A combination of high seed loads and cow aggregated dung deposits likely drive the spatial structuring of seed dispersal observed in Martinazo. Additionally, the fact that *Plantaginaceae* and *Cyperaceae*, the seed families associated with Martinazo cattle, exhibit significant spatial dispersal patterns, very similar to those of total seeds, further reinforce the role of cow in generating spatial structuring of seed dispersal. Horses, like cattle, are large-bodied ungulates capable of exerting substantial influence on ecosystem structure and function. Evaluating the potential role of horses in the formation of spatial patterns is therefore of considerable interest. However, the current dataset contains too few fecal samples with seeds dispersed by horses to permit a robust assessment of their contribution to generate spatial patterns. A similar limitation applies to wild boars, for which insufficient data precludes a reliable evaluation of their influence on spatial pattern formation.

Differential spatial patterns of seed dispersal between cattle and deer, the two ungulates that diverged more in seed families dispersed in Martinazo, point to their complementary role in the dispersal process. Studies on complementarity between co-ocurring wild and domestic ungulates, specifically deer and cattle, have traditionally focused on selective grazing [68,69]. A recent study [46] assessed how temporal variability in plant productivity and livestock abundance (cattle and horses) influences the population dynamics of red deer and fallow deer in Doñana National Park. They concluded that domestic ungulates positively affect deer population density at low to intermediate livestock abundances, whereas negative effects emerge at high abundances. Thus, our results demonstrate complementarity in seed dispersal between cattle and deer, revealing an important ecological role shared by these potentially competing wild and domestic ungulates in the Mediterranean grasslands of Doñana National Park.

### 4.4. Conclusions

Seed dispersal by different ungulate species in the studied Mediterranean grasslands was neither random nor redundant. Instead, it is shaped by complex interactions between disperser identity, plant community composition, and landscape heterogeneity. The findings presented here shed light on the ecological role of multi-species communities of wild and domestic ungulates, their complementarity as dispersers and the relevance of considering functional differences among herbivores when designing conservation and restoration strategies. Thus, they provide valuable insights for decision-making on the management of these large herbivores in protected areas under current or future environmental challenges.

## Supporting information

S1 Table Values used in estimating the dry weight of fecal units and the bibliographic data source. This table provides information on feces fresh weight (FW), feces water content, and source for the ungulate dispersers occurring in the study sites. It also includes the dry weight (DW) of feces per defecation, calculated from the previous two values.
(DOCX)

S2 Table Taxonomic composition of the seeds dispersed in the study samples. This table provides information on the number of seeds at different taxonomic levels (family, genus, species, not identified) in ungulate fecal samples from Matasgordas and Martinazo. Frequency of occurrence (number of samples where the seed species was present out of 114 samples in Matasgordas and 119 samples in Martinazo). Maximum number of seeds found in a sample, and Cumulative number of seeds in all samples from each site.
(DOCX)

S1 Fig The r-mark correlation function, Schlather’s correlation function and density correlation function. Interpretations and graphical explanations. Figures and captions on the meaning of different spatial correlation functions.
(DOCX)
