# Supplementary material for: Spatial pattern of herbaceous seed dispersal by ungulates in grasslands of Doñana, SW Spain
Source: PLoS One. 2026 Mar 9;21(3):e0327616. doi: 10.1371/journal.pone.0327616 (PMC12970880; doi:10.1371/journal.pone.0327616)
Supplement: S1 Table — This table provides information on feces fresh weight (FW), feces water content, and source for the ungulate dispersers occurring in the study sites. It also includes the dry weight (DW) of feces per defecation, calculated from the previous two values. (DOCX) [file pone.0327616.s001.docx]

**SUPPLEMENTARY MATERIAL.**

**Article title:** Spatial pattern of herbaceous seed dispersal by ungulates in Doñana National Park grasslands, SW Spain.

**Journal name**: PLOS ONE

**Authors names**: María José Leiva and Jose María Fedriani

**Values used in estimating the dry weight of fecal units and source of data**

**S1 Table**  Feces fresh weight (FW) and feces water content in the ungulate dispersers occurring in the study sites. Values are the average from different literature sources (superscript numbers). The dry weight (DW) of feces per defecation is calculated from the previous two values.

| Disperser | Feces FW per defecation (g) | Feces water content (%) | Feces DW per defecation (g) |
| --- | --- | --- | --- |
| Deer | 62^1,2,3^ | 20 ^4^ | 49.6 |
| Cow | 2100 ^5,6^ | 80 ^5,7^ | 630 |
| Horses | 1500 ^8,9^ | 80 ^10^ | 300 |
| Wild board | 43^1,2^ | 70 ^11^ | 12.9 |

1 Picard M, Papaïx J, Gosselin F, Picot D, Bideau E, Baltzinger C. Temporal dynamics of seed excretion by wild ungulates: implications for plant dispersal. Ecology and Evolution 2015; 5:2621–2632. <https://doi.org/10.1002/ece3.1512>.

2 Picard M, Chevalier R, Barrier R, Boscardin Y, Baltzinger C. Functional traits of seeds dispersed through endozoochory by native forest ungulates. Journal of Vegetation Science 2016; 27: 987–998. <https://doi.org/10.1111/jvs.12418>.

3 Monfort SL, Harvey E, Geurts L, Padilla L, Simmons HA, Williamson LR. et al. Urinary 3 alpha,17beta-androstanediol glucuronide is a measure of androgenic status in Eld's deer stags (Cervus eldi thamin) Biology of Reproduction 1995; 53: 700-706. <https://doi.org/10.1095/biolreprod53.3.700>.

4 Bond T, Sear D, Sykes T. Estimating the contribution of in-stream cattle faeces deposits to nutrient loading in an English Chalk stream. Agricultural Water Management 2014; 131:156-162. <https://doi.org/10.1016/j.agwat.2013.08.015>.

5 Pan S, Wang G, Fan Y, Wang X, Liu J, Guo M, et al. Enhancing the compost maturation of deer manure and corn straw by supplementation via black liquor. Heliyon 2023; 9 (2) e13246. <https://doi.org/10.1016/j.heliyon.2023.e13246>

6 Finn JA and Guillet PS. Patch size and colonisation patterns: an experimental analysis using north temperate coprophagous dung beetles. ECOGRAPHY 2000; 23: 315–327.

7 Yoshitake S, Soutome H, Koizumi H. Deposition and decomposition of cattle dung and its impact on soil properties and plant growth in a cool-temperate pasture. Ecol Res 2014; 29: 673–684. <https://doi.org/10.1007/s11284-014-1153-2>

8 Alonso JM, Schmitt FP, Sousa FAL, Rosa GS, Esper CS, Melo Neto GB. Carboxymethylcellulose and psyllium effects in sand output of horses with asymptomatic sand accumulation. Arq. Bras. Med. Vet. Zootec. 2020; 72:1609-1617. <https://doi.org/10.1590/1678-4162-11525>.

9 Bessonnat A, Vanore M. Effect of topical cyclopentolate alone or combined with phenylephrine in healthy horses. Veterinary Ophthalmology 2021; 24:582–590. <https://doi.org/10.1111/vop.12896>.

10 Williams S, Horner J, Orton E, Green M, McMullen S, Mobasheri A et al. Water intake, fecal output and intestinal motility in horses moved from pasture to a stable management regime with controlled exercise. Equine vet J 2015; 47(1): 96–100. <https://doi.org/10.1111%2Fevj.12238>

11 HwangBo J, Hong E-C, Park H-D, Kim D-W, Cho S-B. The Study on the Amount and Major Compositions of Excreta from Swine. *Journal of Animal Science and Technology* 2010; 52(4). <http://dx.doi.org/10.5187/JAST.2010.52.4.319>
