# Supplementary material for: Spatial pattern of herbaceous seed dispersal by ungulates in grasslands of Doñana, SW Spain
Source: PLoS One. 2026 Mar 9;21(3):e0327616. doi: 10.1371/journal.pone.0327616 (PMC12970880; doi:10.1371/journal.pone.0327616)
Supplement: S2 Table — This table provides information on the number of seeds at different taxonomic levels (family, genus, species, not identified) in ungulate fecal samples from Matasgordas and Martinazo. Frequency of occurrence (number of samples where the seed species was present out of 114 samples in Matasgordas and 119 samples in Martinazo). Maximum number of seeds found in a sample, and Cumulative number of seeds in all samples from each site. (DOCX) [file pone.0327616.s002.docx]

SUPPLEMENTARY MATERIAL.

Article title: Spatial pattern of herbaceous seed dispersal by ungulates in Doñana National Park grasslands, SW Spain.

Journal name: PLOS ONE

Authors names: María José Leiva and Jose María Fedriani

**Taxonomic composition of the seeds dispersed in the study samples**

**S2 Table** . Number of seeds at different taxonomic levels (family, genus, species) in samples of Matasgordas and Martinazo. Freq. = Frequency of occurrence (number of samples where the seed species is present out of 114 samples in Matasgordas and 119 samples in Martinazo). Max. = maximum number of seeds found in a sample, Cum. = cumulative number of seeds in all samples from each site. NI = not identified to a higher taxonomic level.

|  | **Matasgordas** | | |  | **Martinazo** | | |
| --- | --- | --- | --- | --- | --- | --- | --- |
|  | **Freq.** | **Max.** | **Cum.** |  | **Freq.** | **Max.** | **Cum.** |
| *Juncaceae* |  |  |  |  |  |  |  |
| *Juncus acutus* | 16 | 517 | 721 |  | 3 | 4 | 7 |
| *Juncus bufonious* | 5 | 46 | 55 |  | 2 | 2 | 3 |
| *Juncus sp.1* | 13 | 2067 | 2265 |  | 6 | 6 | 10 |
| *Fabaceae* |  |  |  |  |  |  |  |
| *Coronilla* repanda | 2 | 1 | 2 |  | 1 | 5 | 5 |
| *Dorycnium rectum* | 4 | 2 | 6 |  | 2 | 3 | 4 |
| *Lotus pedunculatus* | 5 | 11 | 16 |  | 2 | 2 | 3 |
| *Lotus sp* | 1 | 2 | 2 |  | 2 | 2 | 4 |
| *Medicago sp.* | 0 | 0 | 0 |  | 1 | 2 | 2 |
| *Melilotus sp.1* | 0 | 0 | 0 |  | 2 | 3 | 4 |
| *Melilotus sp.2* | 0 | 0 | 0 |  | 1 | 1 | 1 |
| *Ononis broterana* | 5 | 4 | 10 |  | 2 | 1 | 2 |
| *Ononis sp.1* | 2 | 1 | 2 |  | 1 | 1 | 1 |
| *Ononis sp.2* | 0 | 0 | 0 |  | 1 | 2 | 2 |
| *Ornithopus pinnatus* | 2 | 1 | 2 |  | 2 | 10 | 14 |
| *Ornithopus compresus* | 3 | 3 | 5 |  | 0 | 0 | 0 |
| *Trifolium campestre* | 27 | 7 | 88 |  | 2 | 7 | 8 |
| *Trifolium glomeratum* | 20 | 21 | 86 |  | 3 | 12 | 15 |
| *Trifolium istmocarpum* | 22 | 62 | 156 |  | 6 | 7 | 16 |
| *Trifolium squamosum* | 7 | 20 | 41 |  | 4 | 4 | 8 |
| *Trifolium tomentossum* | 4 | 1 | 4 |  | 6 | 10 | 20 |
| Trifolium sp.1 | 4 | 2 | 5 |  | 4 | 2 | 5 |
| Trifolium sp.2 | 1 | 4 | 4 |  | 0 | 0 | 0 |
| *Trifolium sp.3* | 14 | 8 | 46 |  | 2 | 1 | 2 |
| *Trifolium sp.4* | 10 | 5 | 22 |  | 1 | 2 | 2 |
| Other *Fabaceae* | 1 | 2 | 2 |  | 0 | 0 | 0 |
| *Caryophyllaceae* |  |  |  |  |  |  |  |
| *Illecebrum verticillatum* | 0 | 0 | 0 |  | 1 | 2 | 2 |
| *Petrorhagia sp.* | 5 | 7 | 17 |  | 0 | 0 | 0 |
| *Polycarpon tetraphillum* | 1 | 2 | 2 |  | 0 | 0 | 0 |
| *Polycarpon sp.* | 0 | 0 | 0 |  | 1 | 1 | 1 |
| *Silene escabrifolia + S. colorata* | 13 | 10 | 36 |  | 6 | 14 | 41 |
| *Silene gallica* | 20 | 8 | 64 |  | 5 | 11 | 21 |
| *Silene sp.* | 3 | 2 | 4 |  | 2 | 3 | 4 |
| *Spergula arvensis* | 6 | 1 | 6 |  | 29 | 15 | 61 |
| *Spergularia maritima* | 1 | 1 | 1 |  | 2 | 3 | 4 |
| *Spergula sp* | 1 | 1 | 1 |  | 0 | 0 | 0 |
| *Stellaria media* | 6 | 5 | 12 |  | 6 | 12 | 21 |
| Other *Cariophyllaceae* | 1 | 1 | 1 |  | 1 | 1 | 1 |
| *Asteraceae* |  |  |  |  |  |  |  |
| *Andryala integrifolia* | 5 | 20 | 24 |  | 5 | 7 | 14 |
| *Chamaemellum sp.* | 11 | 4 | 15 |  | 1 | 1 | 1 |
| *Hypochaeris sp.* | 1 | 1 | 1 |  | 2 | 1 | 2 |
| *Leontodon salzmannii* | 3 | 2 | 5 |  | 0 | 0 | 0 |
| *Tolpis barbata + T. umbellata* | 7 | 3 | 9 |  | 23 | 18 | 61 |
| Other *Asteraceae 1* | 4 | 1 | 4 |  | 8 | 5 | 12 |
| Other *Asteraceae 2* | 0 | 0 | 0 |  | 1 | 1 | 1 |
| Other *Asteraceae 3* | 2 | 1 | 4 |  | 3 | 1 | 3 |
| *Plantaginaceae* |  |  |  |  |  |  |  |
| *Plantago bellardii + P. lanceolata* | 21 | 18 | 53 |  | 3 | 4 | 7 |
| *Plantago coronopus* | 11 | 4 | 19 |  | 38 | 31 | 229 |
| *Plantago serraria* | 2 | 1 | 2 |  | 20 | 7 | 29 |
| *Plantago* sp.1 | 6 | 3 | 9 |  | 4 | 5 | 10 |
| *Plantago sp.2* | 0 | 0 | 0 |  | 3 | 1 | 3 |
| *Plantago sp.3* | 4 | 3 | 6 |  | 0 | 0 | 0 |
| *Poaceae* |  |  |  |  |  |  |  |
| *Anthoxantum sp.* | 2 | 1 | 2 |  | 3 | 12 | 15 |
| *Bromus hordeaceous* | 4 | 3 | 7 |  | 1 | 1 | 1 |
| *Bromus rígidus* | 0 | 0 | 0 |  | 2 | 4 | 5 |
| *Paspalum vaginatum* | 6 | 10 | 15 |  | 1 | 1 | 1 |
| *Phalaris sp.* | 2 | 4 | 5 |  | 0 | 0 | 0 |
| *Vulpia sp.* | 1 | 1 | 1 |  | 1 | 1 | 1 |
| *Other Poaceae* NI1 | 0 | 0 | 0 |  | 2 | 2 | 3 |
| *Other Poaceae* NI2 | 3 | 2 | 4 |  | 1 | 1 | 1 |
| *Other Poaceae* NI3 | 2 | 4 | 6 |  | 0 | 0 | 0 |
| *Other Poaceae* NI4 | 4 | 6 | 9 |  | 0 | 0 | 0 |
| *Other Poaceae* NI5 | 1 | 1 | 1 |  | 0 | 0 | 0 |
| *Other Poaceae* NI6 | 0 | 0 | 0 |  | 5 | 1 | 5 |
| *Primulaceae* |  |  |  |  |  |  |  |
| *Anagallis sp.* | 2 | 1 | 2 |  | 0 | 0 | 0 |
| *Samolus valerandi* | 10 | 4 | 20 |  | 15 | 16 | 52 |
| *Apiaceae* |  |  |  |  |  |  |  |
| *Ammi viznaga* | 5 | 1 | 5 |  | 2 | 11 | 12 |
| *Apiaceae* NI1 | 0 | 0 | 0 |  | 1 | 1 | 1 |
| *Apiaceae* NI2 | 1 | 1 | 1 |  | 0 | 0 | 0 |
| *Cyperaceae* |  |  |  |  |  |  |  |
| *Cyperus longus + C. rotundus* | 1 | 1 | 1 |  | 9 | 7 | 26 |
| *Eleocharis palustris* | 24 | 4 | 29 |  | 6 | 25 | 49 |
| *Isolepis pseudocetacea* | 2 | 1 | 2 |  | 3 | 9 | 13 |
| *Isolepis cernuus* | 0 | 0 | 0 |  | 4 | 3 | 6 |
| *Schoenoplectus corymbosus* | 5 | 3 | 7 |  | 19 | 18 | 82 |
| *Scirpus cernuus* | 1 | 1 | 1 |  | 0 | 0 | 0 |
| *Cyperaceae* NI1 | 15 | 2 | 17 |  | 37 | 11 | 57 |
| *Cyperaceae* NI2 | 2 | 2 | 3 |  | 0 | 0 | 0 |
| *Cyperaceae* NI3 | 8 | 30 | 69 |  | 15 | 2 | 18 |
| *Polygonaceae* |  |  |  |  |  |  |  |
| *Rumex bucephalophorus+R. acetosella* | 6 | 1 | 6 |  | 4 | 14 | 25 |
| *Rumex pulcher* | 8 | 7 | 23 |  | 1 | 4 | 4 |
| *Chenopodiaceae* |  |  |  |  |  |  |  |
| *Chenopodium album* | 6 | 6 | 15 |  | 0 | 0 | 0 |
| *Ranunculaceae* |  |  |  |  |  |  |  |
| *Anemone sp.* | 0 | 0 | 0 |  | 1 | 1 | 1 |
| *Nigella sp.* | 0 | 0 | 0 |  | 1 | 1 | 1 |
| *Ranunculus longipes + R. trilobus* | 20 | 17 | 62 |  | 27 | 20 | 136 |
| *Ranunculus macrophillus+ R. bulbosus* | 4 | 3 | 6 |  | 1 | 2 | 2 |
| *Ranunculus* *Ophioglossifolious* | 1 | 1 | 1 |  | 11 | 10 | 32 |
| *Ranunculus peltatus* | 1 | 2 | 2 |  | 2 | 1 | 2 |
| *Ranunculus sp.1* | 19 | 12 | 76 |  | 6 | 14 | 28 |
| *Ranunculus sp.2* | 1 | 1 | 1 |  | 0 | 0 | 0 |
| *Cistaceae* |  |  |  |  |  |  |  |
| *Helianthemun sp.* | 0 | 0 | 0 |  | 2 | 1 | 2 |
| *Tuberaria sp.* | 12 | 40 | 82 |  | 1 | 2 | 2 |
| *Malvaceae* |  |  |  |  |  |  |  |
| *Malva hispanica* | 1 | 1 | 1 |  | 0 | 0 | 0 |
| *Malva parviflora* | 0 | 0 | 0 |  | 1 | 2 | 2 |
| *Malva sp.* | 2 | 1 | 2 |  | 0 | 0 | 0 |
| *Euphorbiaceae* |  |  |  |  |  |  |  |
| *Euphorbia pterococca + E. exigua* | 0 | 0 | 0 |  | 1 | 1 | 1 |
| *Mercurialis sp.* | 2 | 1 | 2 |  | 0 | 0 | 0 |
| *Borraginaceae* |  |  |  |  |  |  |  |
| *Borrago officinalis* | 0 | 0 | 0 |  | 1 | 1 | 1 |
| *Echium sp.* | 3 | 1 | 3 |  | 0 | 0 | 0 |
| *Myosotis sp.* | 0 | 0 | 0 |  | 8 | 9 | 27 |
| *Borraginaceae* NI1 | 0 | 0 | 0 |  | 1 | 1 | 1 |
| *Borraginaceae* NI2 | 0 | 0 | 0 |  | 1 | 1 | 1 |
| *Alismataceae* |  |  |  |  |  |  |  |
| *Alisma lanceolatum* | 0 | 0 | 0 |  | 2 | 3 | 5 |
| *Valerianaceae* |  |  |  |  |  |  |  |
| *Centranthus calcitrapae* | 57 | 139 | 1129 |  | 16 | 7 | 27 |
| *Escrophulariaceae* |  |  |  |  |  |  |  |
| *Linaria sp.* | 1 | 6 | 6 |  | 1 | 2 | 2 |
| *Plumbaginaceae* |  |  |  |  |  |  |  |
| *Armeria sp.* | 1 | 5 | 5 |  | 0 | 0 | 0 |
| *Ericaceae* |  |  |  |  |  |  |  |
| *Erica sp.* | 11 | 6 | 20 |  | 19 | 9 | 41 |
|  |  |  |  |  |  |  |  |
| NOT IDENTIFIED (include 47 morphotypes) | 58 | 12 | 153 |  | 36 | 7 | 80 |
|  |  |  |  |  |  |  |  |
