# Supplementary material for: Spatial pattern of herbaceous seed dispersal by ungulates in grasslands of Doñana, SW Spain
Source: PLoS One. 2026 Mar 9;21(3):e0327616. doi: 10.1371/journal.pone.0327616 (PMC12970880; doi:10.1371/journal.pone.0327616)
Supplement: S1 Fig — Interpretations and graphical explanations. Figures and captions on the meaning of different spatial correlation functions. (DOCX) [file pone.0327616.s003.docx]

**S3 Figures. The r-mark correlation function, Schlather’s correlation function and density correlation function. Interpretations and graphical explanations**


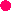

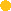


**Example of r-Mark correlation function Kml(r)** for total seeds dispersed in fecal deposits. The function describes the mean number of seeds (mi) in a fecal unit placed at distance *r* of another fecal unit.

Observed function = black solid dots. Expected function under the null model (random distribution of the number of seeds across fecal units) = black line. Simulation envelopes = grey lines which represent the fifth highest and fifth lowest values of the function created by 199 simulations under the null model. X axis corresponds to distance in meters.

A significant function (P=0.005) indicates distance dependence of the seed content in the fecal deposits. In the left part of the graph the observed function reaches values >1 and exceed the upper envelope in the range of distance 2.5 to 3.5 m (pink dot) indicating that at this scale there is a positive effect of feces aggregation (the seeds content of feces that have nearby feces at 2.5 – 3.5 m is on average larger than expected by random). In contrast at distance ranging 22.5 – 24.5 m (yellow dot) the observed function reaches values < 1 and fell below the lowest envelope, indicating a negative effect of feces aggregation (the seeds content of feces that have nearby feces at 22.5 – 24.5 m is on average lower than expected by random).


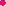


Lowest envelope


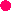


Null model

Highest envelope

Observed Schlather correlation function

**Example of** **Schlather’s correlation function** for total seeds dispersed in fecal deposits. The function describes the spatial covariance in seeds content of two fecal units separated a distance *r*.

Observed function = black solid dots. Expected function under the null model (random distribution of the number of seeds across fecal units) = black line. Simulation envelopes = grey lines which represent the fifth highest and fifth lowest values of the function created by 199 simulations under the null model. X axis corresponds to distance in meters.

A significant function (P=0.005) indicates distance dependence of seed content in fecal deposits. In the left part of the graph the observed function Schlather’s reaches values >0 and exceed the upper envelope in the range of distance 0.5 to 2.5 m (pink dot) indicating that at this scale there is covariation in the seed content of nearby feces (the similarity in seeds content of feces that have nearby feces at 0.5 – 2.5 m is on average higher than expected by random) and the same is true at distance 34-35.5 m.

Observed Density correlation


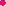

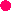

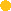


**Example of density correlation function** for total seeds dispersed in fecal deposits.

The function assesses the correlation between the number of seeds and the number of nearby feces deposits located at a distance r.

Observed function = black solid dots. Expected function under the null model (random distribution of the number of seeds across fecal units) = black line. Simulation envelopes = grey lines which represent the fifth highest and fifth lowest values of the function created by 199 simulations under the null model. X axis corresponds to distance in meters.

A marginally significant function (P=0.055) indicates certain distance dependence of seed content in fecal deposits and density of nearby feces units. The observed function reaches values >0 in the left part of the graph where it exceeds the upper envelope in the range of distance 0.5 to 3.5 m (pink dot) indicating positive correlation among seed content and density of nearby feces and the same is true at distance 48m. In contrast, at distance ranging 23.5 – 25.5 m the observed function reaches values < 0 and fell below the lowest envelope (yellow dot), indicating a negative effect of feces density on seed content.
